# Lightweight dense video captioning with cross-modal attention and knowledge-enhanced unbiased scene graph

**DOI:** 10.1007/s40747-023-00998-5

**Published:** 2023-02-24

**Authors:** Shixing Han, Jin Liu, Jinyingming Zhang, Peizhu Gong, Xiliang Zhang, Huihua He

**Affiliations:** 1 College of Information Engineering, Shanghai Maritime University, Shanghai, 201306 China; 2 College of Early Childhood Education, Shanghai Normal University, Shanghai, 200234 China

**Keywords:** Dense video captioning, Cross-modal attention, Commonsense reasoning, Heterogeneous knowledge, Unbiased scene graph

## Abstract

Dense video captioning (DVC) aims at generating description for each scene in a video. Despite attractive progress for this task, previous works usually only concentrate on exploiting visual features while neglecting audio information in the video, resulting in inaccurate scene event location. In this article, we propose a novel DVC model named CMCR, which is mainly composed of a cross-modal processing (CM) module and a commonsense reasoning (CR) module. CM utilizes a cross-modal attention mechanism to encode data in different modalities. An event refactoring algorithm is proposed to deal with inaccurate event localization caused by overlapping events. Besides, a shared encoder is utilized to reduce model redundancy. CR optimizes the logic of generated captions with both heterogeneous prior knowledge and entities’ association reasoning achieved by building a knowledge-enhanced unbiased scene graph. Extensive experiments are conducted on ActivityNet Captions dataset, the results demonstrate that our model achieves better performance than state-of-the-art methods. To better understand the performance achieved by CMCR, we also apply ablation experiments to analyze the contributions of different modules.

## Introduction

Video captioning (VC), also known as automatic natural sentence description of videos, is crucial for connecting vision and language. It has plenty of practical applications, e.g., human–robot interaction, video indexing and describing videos for the visually impaired.

Early works [1–4] described a video content with only one sentence, which might be too sparse for videos. For example, one video has the caption “someone sliced the potatoes with a knife, chopped the onions into pieces, and put the onions and potatoes into the pot.”. Machine may generate “someone is cooking”. While correct, the description is not specific enough, and not the answer we want. In real world, a video usually contains multiple events, so the generated caption needs to be able to describe each event. Thus, Krishna et al. [5] pioneered a new concept called dense video captioning. DVC aims to localize events from an untrimmed video and describe them using natural language. Another contribution of Krishna et al. is to construct the ActivityNet Captions dataset, and annotate each time-series localization of video with multiple text sentences. They localize events based on the action extraction method DAP [6]. Each event is then encoded using an LSTM to generate a description. However, they failed to achieve satisfactory results on ActivityNet Captions dataset. After the concept of DVC was proposed, many DVC methods [7–10] have been proposed. Nevertheless, due to multiple events in long videos and overlapping regions, it is difficult to localize events accurately. This problem has also consistently limited the overall accuracy of DVC. To this end, we propose a novel event refactoring algorithm to filter and reconstruct captions. Furthermore, most related methods [7–10] only use a single modality to analyze the input video without exploiting other modalities, limiting the descriptive power of the model. In order to solve this problem, we propose a cross-modal attention mechanism to mix visual and audio bi-modal data in video to improve model performance. Recently, Iashin et al. [11] proposed a novel dense video captioning framework, which is based on the transformer [12]. This framework uses the pre-trained VGGish [13], I3D [14] and word embedding model to extract features from audio, visual and text, respectively, and then send the features to three transformers for encoding and decoding, incurring expanded redundancy of the model parameters. To avoid this problem, in our model, event proposal generator and caption generator share the same cross-modal encoder, greatly reduces redundant parameters.

Moreover, existing DVC methods focus on improving the accuracy of localizing events, the ability of generating captions is insufficient. This is due to two reasons. First, most existing methods directly convert visual features into textual captions, and few of them utilize high-level semantics, relations, and constraints between entities, leaving the visual information not fully exploited. Second, for some methods [15–18] that use common sense reasoning, when extracting prior knowledge, they take co-occurrence relationships in visual information as causal relationships between entities, resulting in biased prior knowledge. Therefore, in CMCR, we propose a commonsense reasoning (CR) module to optimize the captions. CR uses an unbiased scene graph to deeply mine the associations between entities, and “causal intervention” [19] is adopted when extracting prior knowledge. This makes the model more focused on the causal relationships between entities and extracts unbiased features.

Our main contributions are summarized as follows:

1. We propose a cross-modal processing (CM) module. It improves events localization accuracy, and greatly reduces size of the overall model.
2. A commonsense reasoning (CR) module is proposed to optimize the logic of generated captions with both heterogeneous prior knowledge and entities’ association reasoning achieved by building a knowledge-enhanced unbiased scene graph.
3. Extensive experiments are conducted on the ActivityNet Captions dataset, the results demonstrate that CMCR achieves better performance than state-of-the-art methods.

## Related work

### Dense video captioning

DVC is a further refinement of common video captioning. Krishna et al. [5], inspired by the dense image captioning task, first proposed the DVC problem. They used a long-short-term memory (LSTM) network to encode context and generate captions to deal with it. Anderson et al. [20] introduced the idea of coherent captioning by observing the overall context and optimizing two-level rewards, further developing the concept of context-awareness. They use the SST [21] to generate proposals and use pointer networks [22] to distill proposal candidates. Based on the SST, Wang et al. [9] proposed a Bi-SST method. To obtain the corresponding context, Bi-SST applied LSTM to encode visual features in both the past and future directions, and then combined them with visual data as the model’s input. Another research route is based on weak supervision, which is designed to solve that problem of time-consuming dataset annotation. Duan et al. [10] proposed an auto-encoder to generate proposals and then describe them in a cycle-consistent manner, while being supervised only with a set of non-localized captions.

The above works are designed for visual modality only, so the valuable information captured in the video, such as the interaction between subject and object, is very limited. Therefore, we believe that information from different modalities may benefit dense video captioning.

### Multi-modal dense video captioning

Many recent deep learning-based works [23–26] make use of multi-modal data to improve the performance in a variety of tasks. It is reasonable to assume that, besides visual information, video understanding might benefit from the cues contained in other modalities like audio [24], speech [25], or both [11, 17]. Rahman et al. [24] first incorporated the audio modalities into dense video captioning. They used the concept of cycle-consistency from [12] and combined information from multiple modalities using multi-modal Tucker decomposition [27] before passing it to a GRU-based caption decoder [28]. Hessel et al. [25] applied transformer [12] to encode video frames and speech segments in videos, and evaluated it on YouCook2. While their models achieved good results, they were constrained to a certain application domain. The method is difficult to produce satisfactory results in other domains of real-world videos. Luo et al. [17] utilized input video and corresponding ASR transcript pairs to train their DVC model, combining video and text feature via self-supervision techniques. Unlike other methods, Iashin et al. [11] use three different modalities. Specifically, they extract features from audio, image, and text by using pre-trained VGGish [13], I3D [14], and word embedding models, and then feed the features to three transformers for encoding and decoding. It can be found that the captions generated are more accurate through the mutual validation and complementarity of the multi-modal data.

### Commonsense reasoning

Commonsense is the everyday consensus that exists generally among people in a social environment. By using commonsense reasoning, we can avoid as much as possible the “cognitive errors” made by machines. Currently, several works [7, 15, 17, 26, 29, 30] use commonsense as prior knowledge for scene understanding tasks. In [15], the scene description graph of an image was obtained using commonsense reasoning, and the graph was directly transformed into a sentence using a template-based language model. Different from these methods that directly extracted explicit semantic concepts from external knowledge, Hou et al. [18] utilized joint common sense and relational reasoning to infer semantic relations, addressing the “hallucinating” [17] problem. Zhou et al. [31] used large-scale commonsense knowledge in an open-domain dialogue generation model to strengthen the model’s understanding ability. Wang et al. [29] proposed a novel unsupervised feature representation method to serve as an improved visual region encoder for high-level tasks. It employed “causal intervention” to learn causal associations between entities. This concept works well on a number of datasets. In this paper, we use commonsense reasoning to construct unbiased scene graph that strengthen the causal associations between entities in complex scenarios.

## Methodology

**Fig. 1:**
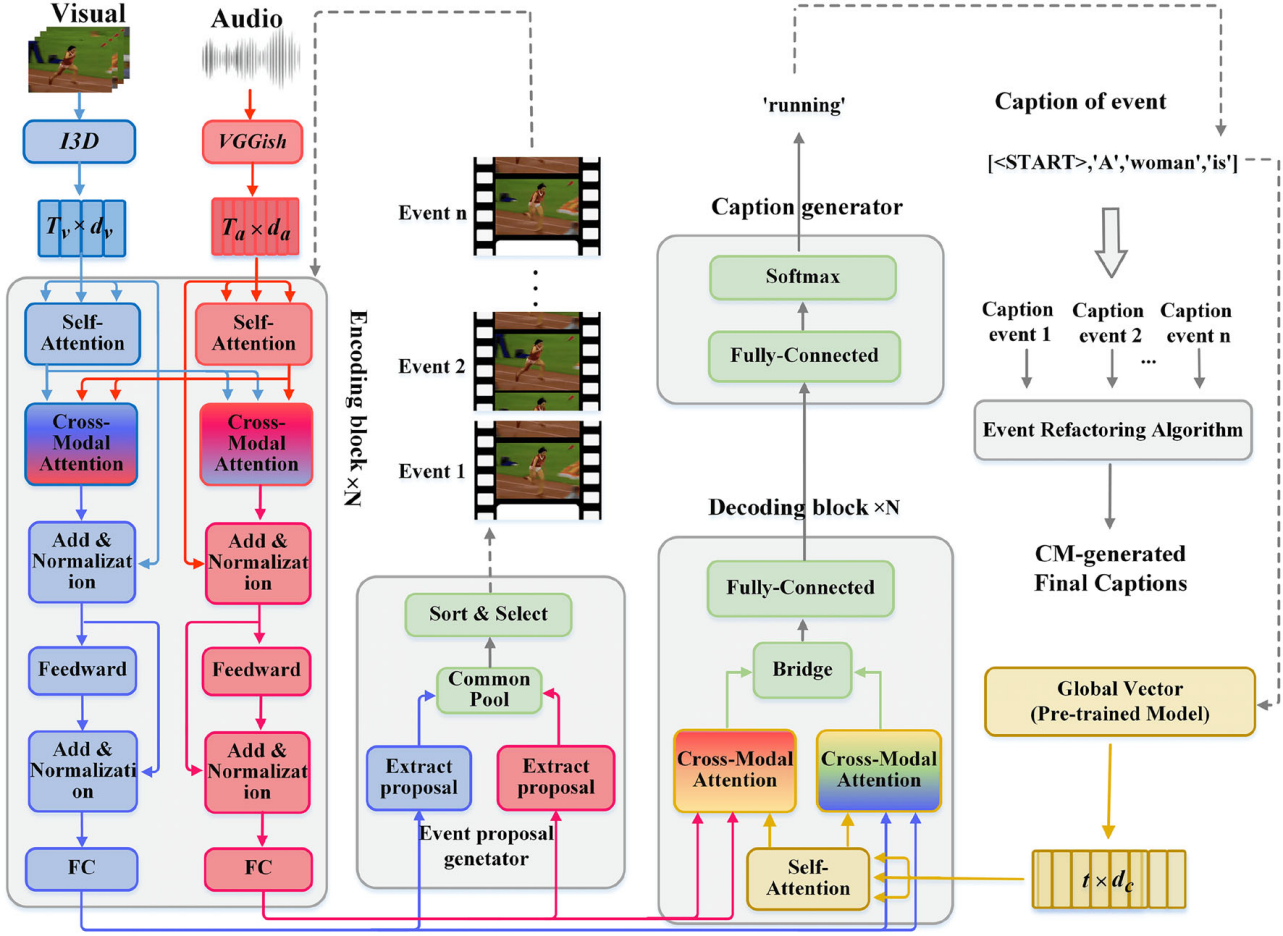
The overall architecture of CM

### Cross-modal processing

The Cross-Modal processing (CM) module is proposed to solve the problem of inaccurate localization events and reduces the model’s redundant parameters, as illustrated in Fig. 1. The following is a brief description of the CM processing. (1) Through the pre-trained I3D and VGGish, the model extracts visual and audio features. (2) The encoder takes features of various modalities as input. (3) The obtained cross-modal features are fed into the event proposal generator to localize proposals. (4) The proposals are fed into the encoder for re-encoding to obtain more detailed features. (5) The decoder fuses more detailed cross-modal features with GloVe-extracted [32] text features, then decodes them into a text sequence. (6) Finally, we propose an event refactoring algorithm that filters and recombines candidate caption sets for overlapping events. CM will eventually generate a video caption that is logical.

#### Cross-modal encoder

**Table 1:** Descriptions of some symbols in cross-modal encoder

Symbol	Description
${a}_{{n} - 1}^\mathrm{{fc}}$	The output of audio sequence on the previous encoding block
${v}_{{n} - 1}^\mathrm{{fc}}$	The output of visual sequence on the previous encoding block
${a}_{{n}}^\mathrm{{self}}$	The output of audio sequence on self-attention layer
${v}_{{n}}^\mathrm{{self}}$	The output of visual sequences on self-attention layer
${a}_{{n}}^\mathrm{{cm}}$	The output of audio sequence on the cross-modal attention fusion layer
${v}_{{n}}^\mathrm{{cm}}$	The output of visual sequence on the cross-modal attention fusion layer
${a}_{{n}}^\mathrm{{fc}}$	The output of audio sequences of the current encoding block
${v}_{{n}}^\mathrm{{fc}}$	The output of visual sequences of the current encoding block

As a first step, we model the different modal information contained in the video source. In this paper, the pre-trained I3D [14] and VGGish [13] are chosen to model dynamic visual and audio information respectively. The dimensions $d_\mathrm{{v}}$ and $d_\mathrm{{a}}$ of visual and audio features are set to 1024 and 128. The features are then fed into the cross-modal encoder. For the cross-modal encoder, we not only encode the information of the different modalities, but most notably by reusing the encoder, we greatly reduce the the number of redundant parameters in the model. It consists of *N* encoding blocks, each of which contains two streams: visual and audio features. In addition, each block has four layers: self-attention, cross-modal attention fusion, feed-forward, and fully connected. Notably, the self-attention layer adopts a multi-head attention mechanism. After encoding, audio-attended visual features and visual-attended audio features are obtained.

The following formulas can be used to summarize the encoder:

1 $$\begin{aligned}{} & {} a_{n}^\mathrm{{self}} = \textrm{MultiHeadAttention}\left( {{W_\mathrm{{a}}^{Q}a}_{n - 1}^\mathrm{{fc}},W_\mathrm{{a}}^{K}a_{n - 1}^\mathrm{{fc}},W_\mathrm{{a}}^{V}a_{n - 1}^\mathrm{{fc}}} \right) \nonumber \\ \end{aligned}$$

2 $$\begin{aligned}{} & {} v_{n}^\mathrm{{self}} = \textrm{MultiHeadAttention}\left( {{W_\mathrm{{v}}^{Q}v}_{n - 1}^\mathrm{{fc}},W_\mathrm{{v}}^{K}v_{n - 1}^\mathrm{{fc}},W_\mathrm{{v}}^{V}v_{n - 1}^\mathrm{{fc}}} \right) \nonumber \\ \end{aligned}$$

3 $$\begin{aligned}{} & {} a_{n}^\mathrm{{cm}} = \textrm{MultiHeadAttention}\left( {{W_\mathrm{{a}}^{Q}a}_{n}^\mathrm{{self}},W_\mathrm{{a}}^{K}v_{n}^\mathrm{{self}},W_\mathrm{{a}}^{V}v_{n}^\mathrm{{self}}} \right) \nonumber \\ \end{aligned}$$

4 $$\begin{aligned}{} & {} v_{n}^\mathrm{{cm}} = \textrm{MultiHeadAttention}\left( {{W_\mathrm{{v}}^{Q}v}_{n}^\mathrm{{self}},W_\mathrm{{v}}^{K}a_{n}^\mathrm{{self}},W_\mathrm{{v}}^{V}a_{n}^\mathrm{{self}}} \right) \nonumber \\ \end{aligned}$$

5 $$\begin{aligned}{} & {} a_{n} = \textrm{LayerNorm}\left( {a_{n}^\mathrm{{cm}} + a_{n - 1}^\mathrm{{fc}}} \right) \end{aligned}$$

6 $$\begin{aligned}{} & {} v_{n} = \textrm{LayerNorm}\left( {v_{n}^\mathrm{{cm}} + v_{n - 1}^\mathrm{{fc}}} \right) \end{aligned}$$

7 $$\begin{aligned}{} & {} a_{n}^{,} = {\textrm{LayerNorm}\left( a \right. }_{n} + \textrm{feedforward}\left( a_{n} \right) ) \end{aligned}$$

8 $$\begin{aligned}{} & {} v_{n}^{,} = {\textrm{LayerNorm}\left( v \right. }_{n} + \textrm{feedforward}\left( v_{n} \right) ) \end{aligned}$$

9 $$\begin{aligned}{} & {} a_{n}^\mathrm{{fc}} = \mathrm{{FC}}\left( a_{n}^{,} \right) \end{aligned}$$

10 $$\begin{aligned}{} & {} v_{n}^\mathrm{{fc}} = \mathrm{{FC}}\left( v_{n}^{,} \right) , \end{aligned}$$

where $Q \in R^{T_{Q} \times D_{Q}}$,$K \in R^{T_{K} \times D_{K}}$,$V \in R^{T_{K} \times D_{K}}$,$W_{i}^{*} \in R^{D_{*} \times D_{s}}$, the dimensions of *K* and *V* are consistent. $W_i^*$ transforms the input vector into a $D_\mathrm{{s}}$-dimension space. $D_\mathrm{{s}} = \frac{D_{Q}}{h}$, *h* is the number of heads in the multi-head attention mechanism. The description of other symbols can be found in Table 1.

While localizing events and generating captions, the event proposal generator and the caption generator reuse the same cross-modal encoder. This is why the cross-modal encoder is thought to effectively reduce the number of parameters in the model, making it more lightweight.

#### Event proposal generator

**Fig. 2:**
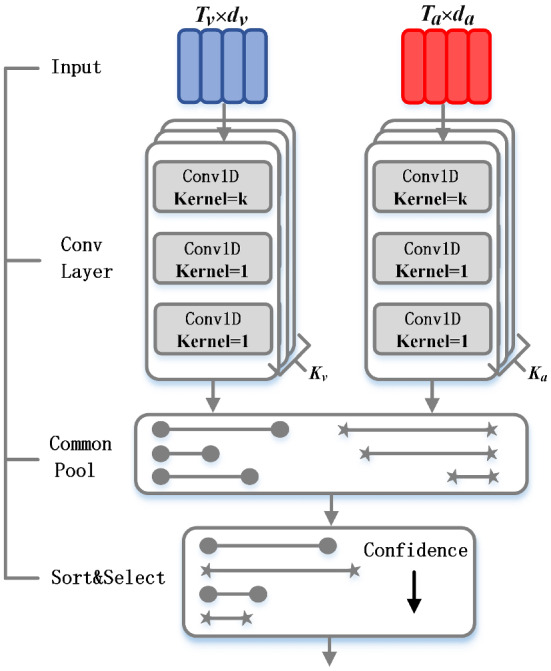
The architecture of event proposal generator

The event proposal generator creates a set of proposals for a video, as shown in Fig. 2. It takes the audio-attended visual (size is $T_\mathrm{{v}} \times d_\mathrm{{v}}$) and visual-attended audio features (size is $T_\mathrm{{a}} \times d_\mathrm{{a}}$) from the cross-modal encoder as input. The Conv layer can extract features and make predictions at each timestamp on the interval [1, *T*]. Specifically, the Conv layer is a fully convolutional network containing three layers. The 1st Conv sub-layer has a kernel size *k* while in the 2nd and the 3rd the kernel size is 1.

Temporal boundaries and confidence for a proposal are obtained using three values which were predicted by the Conv layer: central time point of the proposal (center), proposal duration (length) and confidence score (confidence).

11 $$\begin{aligned} \mathrm{{center}}= & {} p + \sigma (c) \end{aligned}$$

12 $$\begin{aligned} \mathrm{{length}}= & {} \mathrm{{anchor}} \cdot e \end{aligned}$$

13 $$\begin{aligned} \mathrm{{confidence}}= & {} \sigma (o) \end{aligned}$$

where $\sigma ( \cdot )$ is sigmoid function which bounds the values into [0, 1] interval, *p* is the center point, *e* is the zoom factor.

Different *k* will make the convolution kernel have different receptive fields. To capture proposals with varying durations dynamically, the audio and visual features must be fully convoluted $K_\mathrm{{v}}$ and $K_\mathrm{{a}}\left( K_\mathrm{{v}} = K_\mathrm{{a}} \right)$ times, respectively, with different *k* values for the first layer of the convolution kernel in each operation. When performing the *m*-th fully convolution operation, $\varphi$ is used to determine the number of proposals that should be predicted. Because the visual and auditory features have different dimensions $T_\mathrm{{v}}$ and $T_\mathrm{{a}}$, we define $\varphi _\mathrm{{v}}$ and $\varphi _\mathrm{{a}}$ $\left. \left( \varphi \right. _\mathrm{{v}} \ne \varphi _\mathrm{{a}} \right)$ to bring $T_\mathrm{{v}} \cdot |\varphi _\mathrm{{v}} |$ close to $T_\mathrm{{a}} \cdot |\varphi _\mathrm{{a}} |$, ensuring that the two streams generate the same number of proposals. The obtained proposals are fed to the common pool, and the top-100 proposals are selected based on the confidence score. The selected proposals are then clustered using K-Means algorithm, and each cluster’s anchor is selected to form a proposal set. Note that the proposal set’s features will be fed back into the cross-modal encoder for re-encoding. The features after re-encoding will be fed to the cross-modal decoder.

#### Cross-modal decoder

Similar to the cross-modal encoder, the cross-modal decoder has *N* decoding blocks, using the re-encoded features as input. We use the Global Vector (GloVe) [32] to convert the word caption into a word embedding. Next, visual feature, audio feature, and word embedding are fused in the cross-modal attention layer of cross-modal decoder. After decoding, the resulting features are fed to the caption generator to generate a text description of each event.

#### Caption generator

The purpose of the caption generator is to model the distribution for the next caption word. It consists of a fully connected layer with softmax activation. Through this layer, we can map the caption features into a dimension corresponding to the size of the vocabulary in the training set. It is worth noting that because the decoding block requires the words generated in the previous time step to help predict the words in the current time step, the generated words will be fed into the decoding block. When decoding the first word, “<START>” will be fed to the decoding block.

#### Event refactoring algorithm

**Fig. 3:**
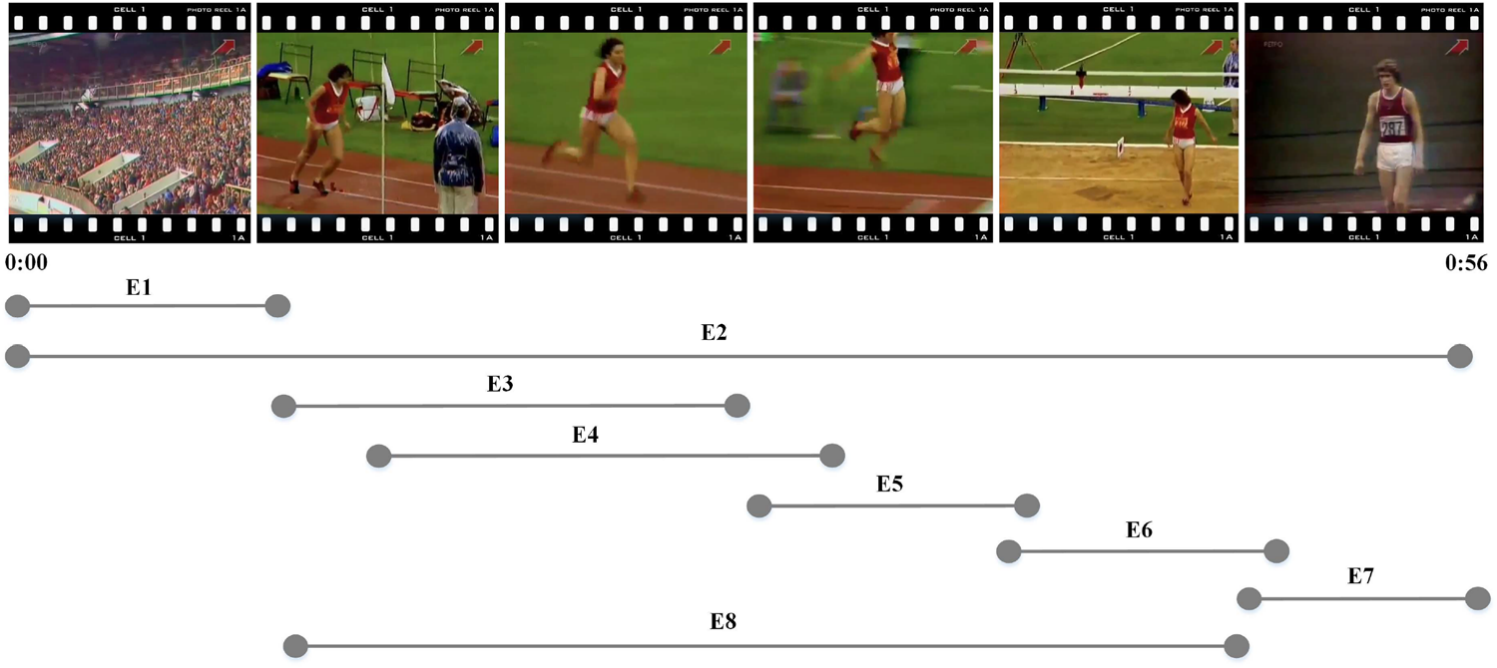
Examples of event proposals in a video. $E_i$ is an event proposal, $i =1, 2,..., 8$


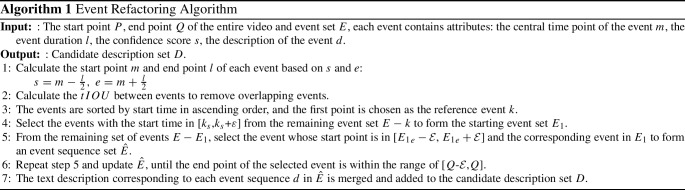


Although the majority of proposals have been filtered out by using K-Means in the event proposal generator, there is still overlap or redundancy between events, as shown in Fig. 3. Therefore, we propose an event refactoring algorithm to de-duplicate and combine the events in the event set, eventually obtaining a candidate description set with real meaning.

The two cases of overlapping events will be explained below. The first case is, for example, $E_3$ and $E_4$. The algorithm uses *tIOU* to remove overlapping events. Unlike *IOU* in computer vision, *tIOU* calculates the degree of overlap between two events.

14 $$\begin{aligned} tIOU = \left\{ \begin{matrix} {~~~~~0~,~~~~if~E_{3} \cap E_{4} = \varnothing } \\ {~\frac{E_{3} \cap E_{4}}{E_{3} \cup E_{4}}~,if~E_{3} \cap E_{4} \ne \varnothing } \\ \end{matrix} \right. \end{aligned}$$

When *tIOU* is greater than the threshold, $E_3$ and $E_4$ are considered as overlapping events. At this time, events with higher confidence scores are prioritized. The threshold is set by us and will be discussed in experiments.

Another case is more common. There may be multiple granularities of events in a full video description, and a high-performance event proposal generator needs to be sensitive to the different granularities of events. For example, in Fig. 3, $E_2$ is a coarse-grained event. $E_1$, $E_3$, $E_5$, $E_6$, $E_7$ are fine-grained events. $E_8$ uses a detection method that falls between coarse and fine-grained. To address this problem, the event refactoring algorithm can consolidate captions of multiple events into a single complete caption while ensuring the temporality and comprehensiveness of the generated caption. The entire workflow of the algorithm is shown in Algorithm 1.

#### Training procedure and details

The CM training procedure is divided into two stages: first, the caption generator is trained with ground truth proposals. Then, the event proposal generator is trained using the cross-modal encoder trained in the first stage. When training the event proposal generator and caption generator, there are some things to keep in mind.

Each time the decoder predicts a word in the training caption generator, it should input the generated word from the previous time step. However, training the event proposal generator in this way would increase the deviation between the training results and the ground truth proposals. Therefore, when training the event proposal generator, each time the decoder predicts a new word during training, the corresponding word in the ground truth caption is taken as the word in the previous time step for the next decoding.

To measure the effect of training, mean square error (MSE) and cross entropy (CE) are used as the loss functions for event detection and localization in event proposal generator, respectively. In addition, we use Kullback–Leibler (KL) divergence as a loss function to train the caption generator, which can measure the difference between predicted and ground-truth captions.

### Commonsense reasoning with knowledge-enhanced unbiased scene graph

**Fig. 4:**
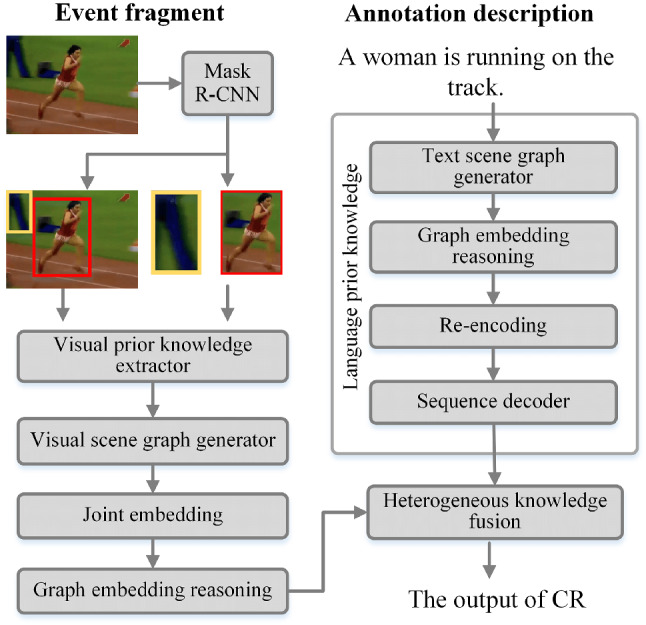
The architecture of CR

CM produced satisfactory results in localizing events, but only generated captions based on visible information and did not make more profound inferences. As a result, we propose a module (CR) to optimize the logic and rationality of the captions generated by CM, as shown in Fig. 4.

CR first utilizes the concept of causal intervention to extract unbiased visual prior knowledge, and then uses counterfactual intervention [19] to construct unbiased scene graphs for semantic representation. The scene graph solves the model’s “shortcut convergence” problem while preserving the visual information’s contextual causal associations. Meanwhile, CR uses the existing annotation description to pre-train a dictionary of prior knowledge of the language, which is then fused with the previously extracted visual knowledge feature after feature embedding. Finally, the fused results are fed into the CM.

#### Acquiring visual prior knowledge

**Visual prior knowledge extractor**

**Fig. 5:**
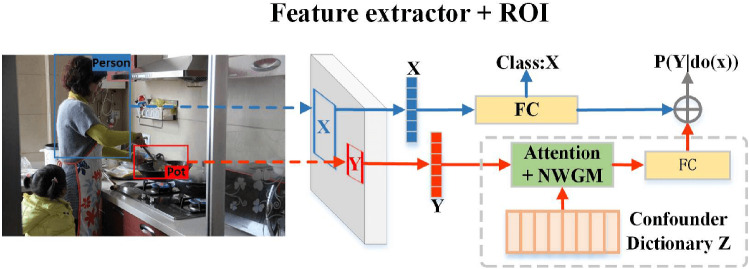
The architecture of visual prior knowledge extractor

**Fig. 6:**
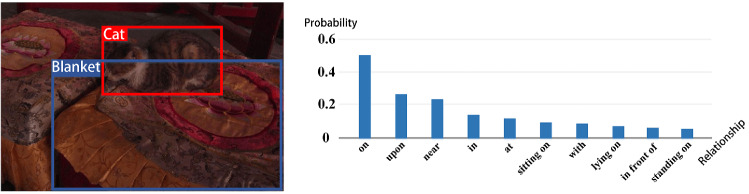
A plot of the predicted probability distribution of a single sample

In causal associations, there are always confounding factors that affect the outcome. For example, “age” is a confounder in the relationship between “corona virus disease” and “mortality”. It brings a bias to the relationship between “corona virus disease” and “mortality”. From the perspective of the second layer of [19], we propose a novel visual prior knowledge extractor (CRvpk) that eliminates the influence of confounding factors. In Fig. 5, the extractor uses a pre-trained mask R-CNN [33] to obtain the ROI of an object from the input image, and then traverses each ROI to perform the following tasks: (1) select an ROI as *X* and use its features to predict the ROI’s class. (2) When *X* is selected, traverse the ROI of other classes in the image as *Y*. The feature corresponding to *Y* and confounders dictionary *Z* [29] are input into the attention layer together and perform to a normalized weighted geometric mean (NWGM) operation. The confounder is the class of each object in the dataset. (3) The weighted summation of (1) and (2) obtains visual features with causal associations.

When constructing an unbiased visual scene graph, CRvpk serves as a pre-trained feature extractor.

**Unbiased visual scene graph generator**

**Fig. 7:**
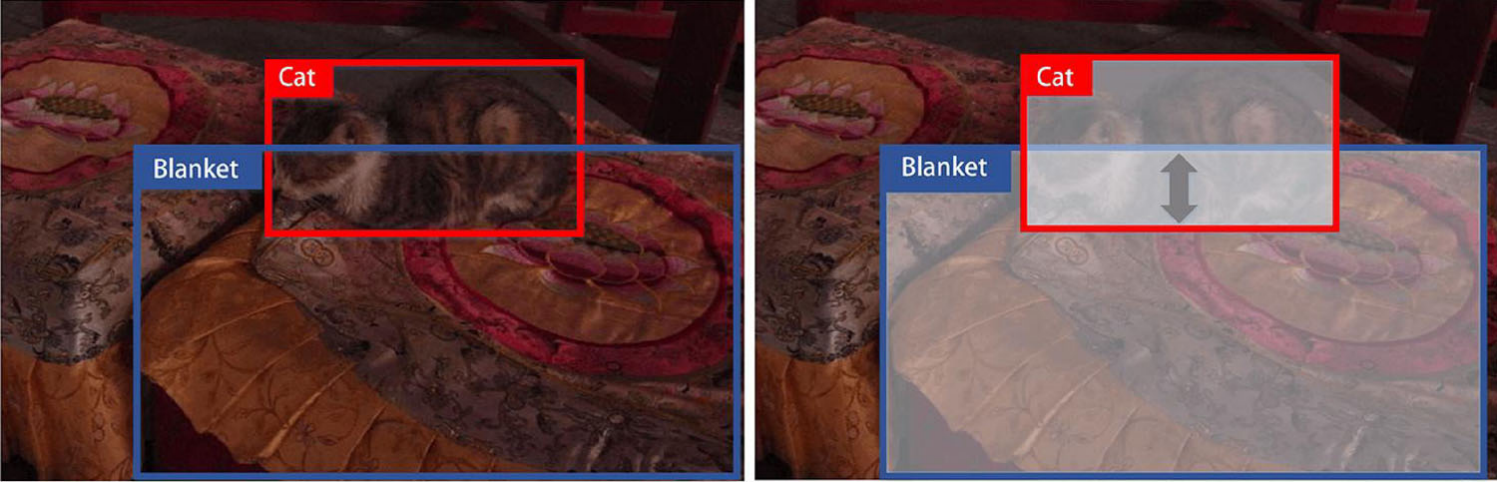
Example diagrams of factual and counterfactual scenarios

**Fig. 8:**
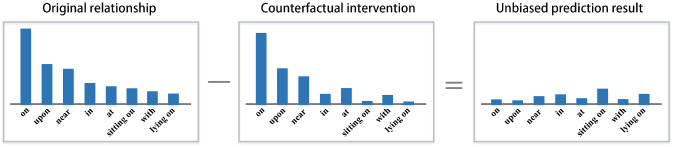
An example of TDE definition applied to scene graph

**Fig. 9:**
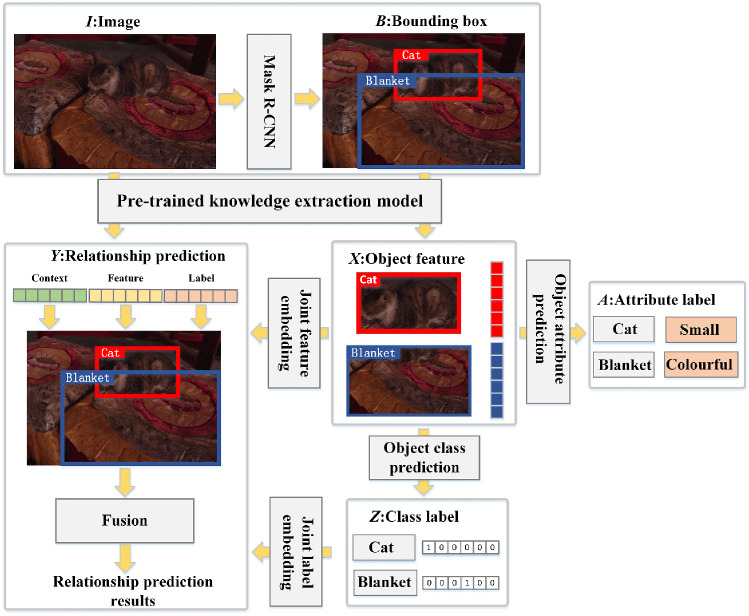
The architecture of unbiased scene graph generator

To extract visual information as priori knowledge for the DVC task, we use scene graphs to obtain visual features. It helps in analyzing and mining the relationships between entities. However, it is believed that when generating scene graph, the Neural Motifs model [34] is more confident in simple relationships and less sensitive in complex relationships, as shown in Fig. 6.

This issue indicates that [34] has a poor understanding of visual information and does not delve deeply into the associations between entities. In other words, it focuses too much on the relative positional relationship between two objects, ignoring the features of the object itself and the causal relationship between objects. To solve this problem, we borrow from counterfactual intervention [19], which requires the model to consider “If the object hadn’t been seen, would the same prediction be made?” before predicting a result. To achieve this, we apply the total direct effect (TDE) mentioned by Tang et al. [35] to the scene graph. Specifically, we use masking to implement counterfactual intervention in Fig. 7, which only focuses on the relative location of objects and masks the features of objects. The distinction between our proposed unbiased scene graph and [35] is shown in Fig. 8. We can get an unbiased prediction result by subtracting the result of the counterfactual intervention from the original relationship.

The implementation of unbiased visual scene graph is based on Neural Motifs, as shown in Fig. 9. *I* is the input image, and $B=\{b_i \}$ is the bounding box of the object in the image.

Through the pre-trained knowledge extraction model (visual prior knowledge extractor), the complete image feature *M* and object feature in the bounding box containing causality $X=\{x_i \}$ can be extracted. On one hand, *X* are decoded in LSTM and cooperated with the fully connected layer to obtain the class label of the object $Z=\{z_i \}$, $z_i$ is expressed by a one-hot vector. On the other hand, *X* are fed to the fully connected layer to get attribute label $A=\{a_i \},i=1,2,...,N$.

**Fig. 10:**
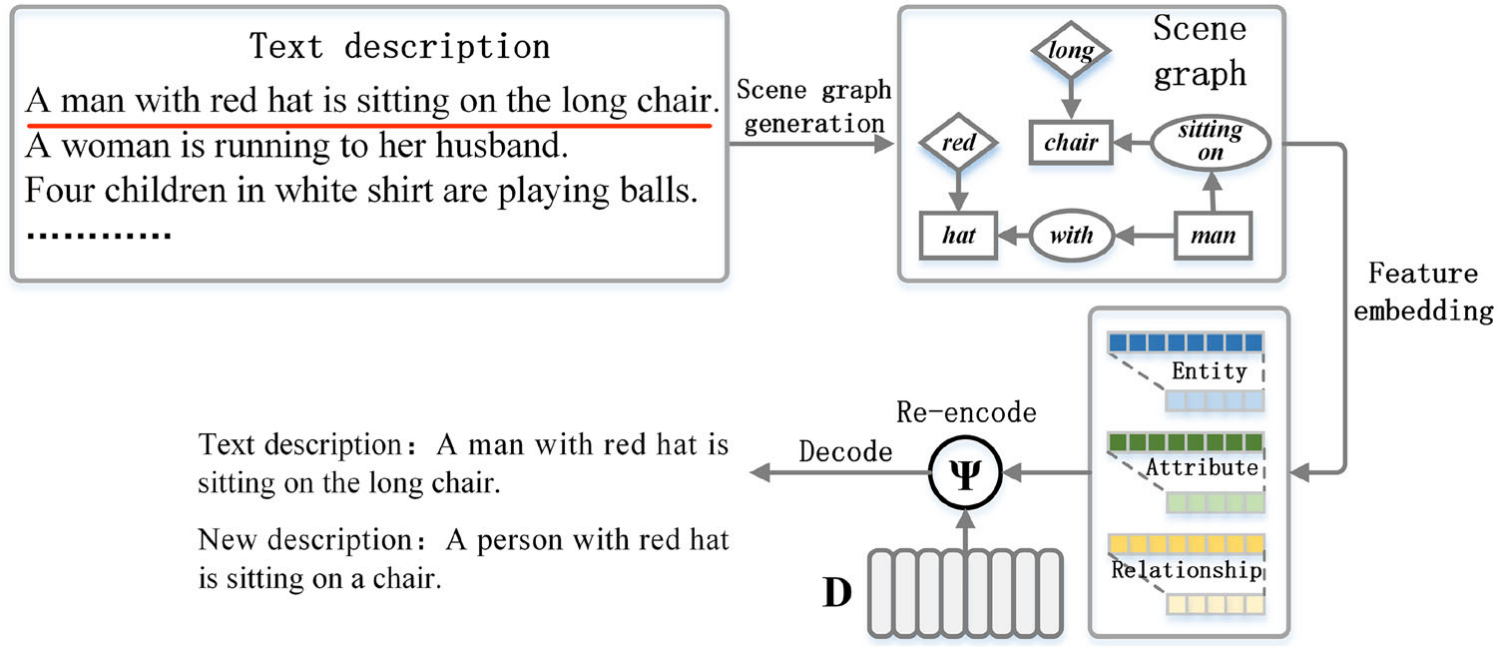
The architecture of language prior knowledge extractor

*X* and *Z* are expressed as feature embedding $X^{'} = \left\{ x_{i}^{'} \right\}$ and label embedding $Z^{'} = \left\{ z_{i}^{'} \right\}$ through joint feature embedding and joint label embedding, respectively. Joint feature embedding combines the features of pairwise objects into a single feature representation. Joint label embedding is used to represent paired labels and provide language prior to the model, i.e., label information. $Z^{'}$ can be computed by

15 $$\begin{aligned} Z^{'} = W^{z}\left\{ z_{i}^{T} \cdot z_{j} \right\} \end{aligned}$$

Then, *M* and *B* are contextually embedded to represent the contextual information between the two bounding boxes. Formally,

16 $$\begin{aligned} V^{'} = \left\{ v_{i}^{'} \right\} = \mathrm{{Conv}}\left( {\mathrm{{RolAlign}}\left( {M,b_{i} \cup b_{j}} \right) } \right) \end{aligned}$$

where Conv is the convolution layer, RolAlign layer aligns the features of original image with the paired bounding box.

In relation prediction, the obtained feature embedding, label embedding and contextual embedding are input to the fusion computing unit,

17 $$\begin{aligned} y_{i} = W_{r}x_{i}^{'} \cdot \sigma \left( {W_{x}x_{i}^{'} + W_\mathrm{{v}}v_{i}^{'} + z_{i}^{'}} \right) \end{aligned}$$

where “$\cdot$” represents the element-wise multiplication of vectors, $\sigma ( \cdot )$ means the sigmoid function. $Y = \left\{ y_{i} \right\}$ is the relationship between the paired objects in the visual information. By semantically modeling of the visual scene graph, we obtain the object feature vector $x_{o_{i}}^{v}$, the attribute feature vector $x_{a_{i}}^{v}$ and the relationship feature vector $x_{r_{ij}}^{v}$. Next, we pad object feature vector with “0”, which is equivalent to masking the object. After that, we repeat the relationship prediction using the visual scene graph and obtain ${{\hat{x}}}_{r_{ij}}^{v}$. $x_{r_{ij}}^{v} - {{\hat{x}}}_{r_{ij}}^{v}$ is the final unbiased association.

#### Acquiring language prior knowledge

Language prior knowledge, in addition to visual prior knowledge, can benefit the model’s understanding of the scene. For example, natural language can enhance semantic representation between entities [36]. Because there is no textual information in the CM input, we decide to pre-train a dictionary of language prior knowledge that can fuse with the visual information, as illustrated in Fig. 10. The concrete implementation is that we use the method of [37] to convert text descriptions into scene graphs and can extract entities, attributes, and relations from the text accurately. Next, *D* and three feature embedding are re-encoded separately. The calculation process of re-encoding $\varphi ( \cdot )$ is as follows:

18 $$\begin{aligned} \varphi \left( {x,D} \right) = {\sum \limits _{l = 1}^{L}{\mathrm{{softmax}}\left( {D^{T}x_{l}} \right) d_{l}}}, \end{aligned}$$

where $d_{l}$ represents the *l*th column vector in *D*. *D* is a predefined initial knowledge dictionary used to encode and embed human language habits (the dimension is $d \times L$).

The re-encoded feature vector is fed into the decoder to reconstruct the text description, and then the learning of the language prior knowledge extractor is supervised by comparing the difference between the reconstructed description and the text description.

#### Heterogeneous knowledge fusion

Existing methods [16–18] for extracting prior knowledge only focus on data from a single modality. To enrich semantic information, we attempt to integrate multi-modal prior knowledge. Before fusion, the object feature vector $$ x_{o_{i}}^{v}$$, attribute feature vector $$x_{a_{i}}^{v}$$, and relationship feature vector $$x_{r_{ij}}^{v}$$ extracted from the visual information should be jointly embedded with their respective label vectors $$x_{o_{i}}^{l}$$, $$x_{a_{i}}^{l}$$ and $$x_{r_{ij}}^{l}$$. The purpose of the joint embedding is to semantically align the feature vector and the label vector, making subsequent fusion relatively easy. The processes of joint embedding can be formulated as:

19 $$\begin{aligned}{} & {} u_{o_{i}} = \mathrm{{ReLU}}\left( {W_{1}^{l}x_{o_{i}}^{l} + W_{1}^{v}x_{o_{i}}^{v}} \right) - \left( {W_{1}^{l}x_{o_{i}}^{l} - W_{1}^{v}x_{o_{i}}^{v}} \right) ^{2} \end{aligned}$$

20 $$\begin{aligned}{} & {} u_{r_{ij}} = \mathrm{{ReLU}}\left( {W_{2}^{l}x_{r_{ij}}^{l} + W_{2}^{v}x_{r_{ij}}^{v}} \right) - \left( {W_{2}^{l}x_{r_{ij}}^{l} - W_{2}^{v}x_{r_{ij}}^{v}} \right) ^{2} \end{aligned}$$

21 $$\begin{aligned}{} & {} u_{a_{i}} = \mathrm{{ReLU}}\left( {W_{3}^{l}x_{a_{i}}^{l} + W_{3}^{v}x_{a_{i}}^{v}} \right) - \left( {W_{3}^{l}x_{a_{i}}^{l} - W_{3}^{v}x_{a_{i}}^{v}} \right) ^{2}, \end{aligned}$$

where $W_i^l$ and $W_i^v$ are the weight parameters acting on the label vector and the feature vector, respectively. $u_{o_{i}}$, $u_{r_{ij}}$ and $u_{a_{i}}$ are the vectors after joint embedding.

Then, graph embedding is applied to $u_{o_{i}}$, $u_{r_{ij}}$ and $u_{a_{i}}$ to reason about visual association. The result of graph embedding is fused with *D* via Eq. (18) to get the output of CR. It will guide the CM in a graph attention network-like manner, as shown in Fig. 4.

### Model integration

**Fig. 11:**
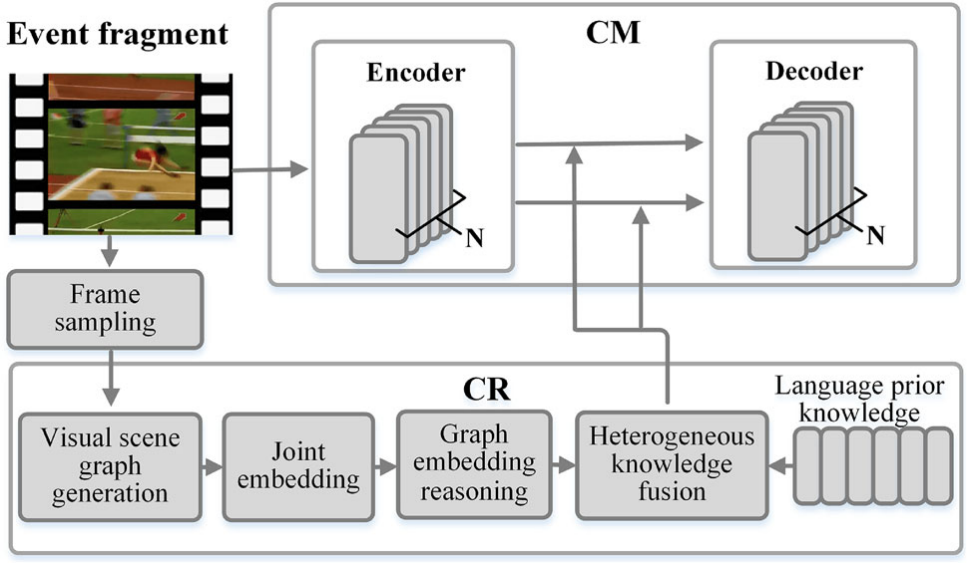
The architecture of CMCR. We only draw the encoding and decoding parts of CM, omitting some modules

Figure 11 illustrates how CR is embedded into the CM. *N* video frames are sampled from the video and fed frame by frame to the CR model to calculate the output *y*. Take the average of on the frame set $$S_{N} = \left\{ {s_{1},s_{2},{\ldots ,s}_{N}} \right\} $$ to get $$\overset{-}{y}$$, and linearly transform $$\overset{-}{y}$$ to get $${\overset{-}{y}}_\mathrm{{v}}^{,}$$, and $${\overset{-}{y}}_\mathrm{{a}}^{,}$$. In CM, the linearly transform makes the dimensions consistent with the dimensions of the visual feature $$v_{n} \in R^{T_\mathrm{{v}} \times d_\mathrm{{v}}}$$ and audio feature $$a_{n} \in R^{T_\mathrm{{a}} \times d_\mathrm{{a}}}$$ output by the encoding block. Fuse $${\overset{-}{y}}_\mathrm{{v}}^{,}$$, $${\overset{-}{y}}_\mathrm{{a}}^{,}$$ with $$ v_{n}$$, $$a_{n}$$ according to the following formula:

22 $$\begin{aligned}{} & {} v_{n}^{,} = v_{n} + W_\mathrm{{v}}{\overset{-}{y}}_\mathrm{{v}}^{,} \end{aligned}$$

23 $$\begin{aligned}{} & {} a_{n}^{,} = a_{n} + W_\mathrm{{a}}{\overset{-}{y}}_\mathrm{{a}}^{,} \end{aligned}$$

Then $v_{n}^{,}$ and $a_{n}^{,}$ are fed to the decoder of CM for decoding to generate captions.

## Experiments

### Dataset and metrics

#### CM

**ActivityNet Captions:** The reason why we only choose ActivityNet Captions is that it contains 100 K dense natural language captions of about 20 K YouTube videos. The dataset is divided into training set, validation set and test set according to the ratio of 2:1:1. More importantly, this dataset not only satisfies the requirement of containing both visual and audio data, but also has a finer granularity of the data. Since the dataset exists in the form of links, and some links are not available, we screened the links one by one and finally retained 89% of videos that contain both visual and audio information. Each video corresponds to multiple text descriptions, and these multiple text descriptions cover 94.6% of the content in the video, with 10% of the temporal information overlapping.

**Table 2:** The impact of the number of encoding blocks or decoding blocks on the number of parameters

*N*	Parameters (million)	Batch size	Training time (h)	GT proposals			Learned proposals		
				B@3	B@4	M	B@3	B@4	M
2	53.55	64	22.3	4.69	2.19	11.08	3.98	1.84	8.93
3	77.26	32	27.5	4.47	2.30	11.16	3.96	1.82	8.87
4	100.98	8	30.7	4.89	2.36	11.42	4.06	1.95	9.11
5	124.70	4	32.1	4.90	2.42	11.63	4.12	1.89	9.13

**Fig. 12:**
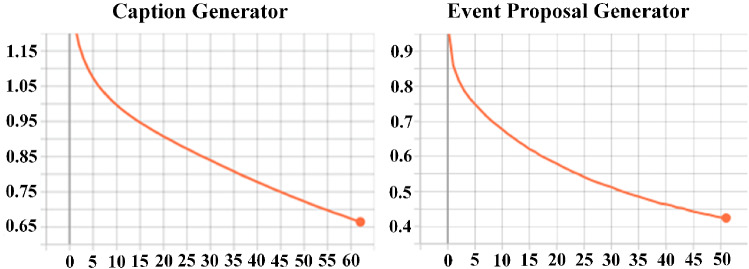
The loss of caption generator and event proposal generator during the training process

**Fig. 13:**
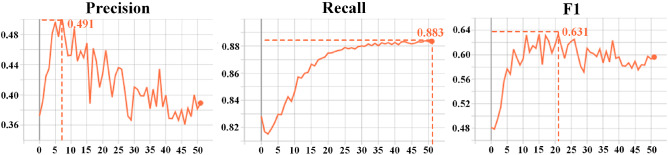
The performance change trend of the event proposal generator in validation process

#### CR

**MS-COCO:** It is a publicly available standard image dataset for classification, detection, segmentation, and description. We use MS-COCO to train the visual prior knowledge extractor and the language prior knowledge extractor. Like other research groups, we split this dataset into training, validation, and testing partitions of 82,783, 40,504, and 40,775 images, respectively.

**Visual Genome 150:** The Visual Genome (VG) comprises 108,077 annotated images. The dataset is divided into training set, validation set, and test set according to the ratio of 3:1:1. Because the object labels in the VG dataset are too confusing, this paper chooses VG150 to train an unbiased visual scene graph. VG150 is a filtered subset of the VG dataset, which contains the 150 most frequently occurring objects and 50 types of relationships in the VG dataset.

#### Metrics

We employ precision, recall, and f1-score to evaluate the performance of localizing events. The performance of generating captions is evaluated by using METEOR [38] and BLEU@3-4 [39].

### Results of CM

#### Experiment details

When training the caption generator and the event proposal generator, we set the batch size to 32 and 16, respectively. In order to make the data form a batch successfully, the caption generator pads all the sequences to the length of the longest sequence in the batch. In the event proposal generator, the visual and audio features are padded to 300 and 800 dimensions, respectively, to form a batch. The reason why 300 and 800 were chosen is that these dimensions are the maximum length that can cover all possible feature lengths in the training set. To avoid interfering with the training results, the padding symbols in the sequence will be masked in the training process.

In the event proposal generator, we set the above-mentioned $\varphi _\mathrm{{v}}$ and $\varphi _\mathrm{{a}}$ to 128 and 48, respectively, and set $K_\mathrm{{v}}$ and $K_\mathrm{{a}}$ to 10 for the number of times the visual and audio features are fully convoluted. The *k* of the 10 convolutional kernels for the visual and audio streams are [5, 13, 23, 35, 51, 69, 91, 121, 161, 211] and [1, 5, 9, 13, 19, 25, 35, 45, 61, 79], respectively.

#### Parameter settings

**Fig. 14:**
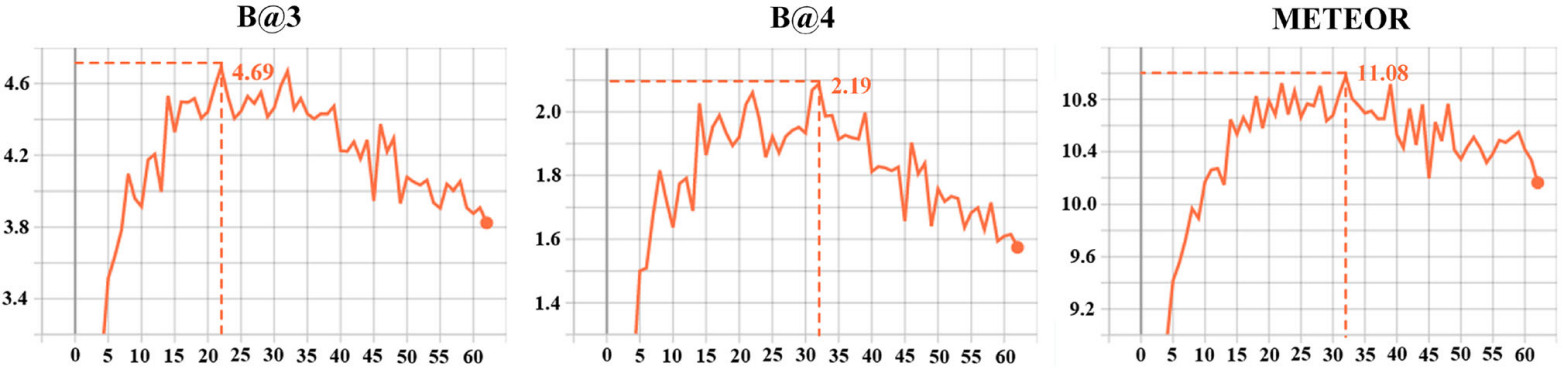
The performance change trend of the caption generator in validation process

This section discusses some parameter settings of CM. First, we discussed the relationship between the parameters of the module, batch size, and performance when a different *N* is selected, as is shown in Table 2. To maintain good performance and avoid too many redundant parameters in the module, we set *N* to 2.

Second, we discuss the epochs of the training event proposal generator and caption generator. In Fig. 12, the loss of caption generator and event proposal generator maintained a steady decline in epochs 62 and 51. Figure 13 shows how the precision, recall, and f1-score of the event proposal generator changed during the validation process. It can be seen that recall is increasing steadily, and precision reached its peak in the 7th epoch. Subsequently, the model may be over-fitted, resulting in a worse validation effect, and the f1-score becomes oscillating. Therefore, on balance, we chose the model obtained from the 21st epoch as the event proposal generator. Figure 14 presents curves depicting the performance of the generated model during the validation process. After analysis, we used the model obtained in the 32nd epoch as the caption generator.

#### Comparison to the state-of-the-art

**Table 3:** Comparison with state-of-the-art models on the dense video captioning task

Method	RL	FD	GT Proposals			Learned proposals		
			B@3	B@4	M	B@3	B@4	M
Li et al. [40]	$\checkmark$	$\checkmark$	4.55	1.62	10.33	2.27	0.73	6.93
Xiong et al. [41]	$\checkmark$	$\checkmark$	–	–	–	2.84	1.24	7.08
Mun et al. [27]	$\checkmark$	$\checkmark$	4.41	1.28	$\mathbf{13.07}$	2.94	0.93	8.82
Krishna et al. [5]	–	$\checkmark$	4.09	1.60	8.88	1.90	0.71	5.69
Li et al. [40]	–	$\checkmark$	4.51	1.71	9.31	2.05	0.74	6.14
Zhou et al. [42]	–	$\checkmark$	5.76	2.71	11.16	2.91	1.44	6.91
Wang et al. [9]	–	$\checkmark$	4.33	2.30	10.89	2.27	1.13	7.84
Mun et al. [27]	–	$\checkmark$	–	–	–	–	–	6.92
Rahman et al. [24]	–	–	3.04	1.46	7.23	1.85	0.90	4.93
Iashin et al. [20]$*$	–	–	4.12	1.81	10.09	2.31	0.92	6.80
iPerceive DVC [41]$*$	–	–	5.23	2.34	11.77	2.59	1.07	7.29
BMT [43]	–	–	4.63	1.99	10.90	3.84	$\mathbf{1.88}$	8.44
Iashin et al. [20]$^\dagger$	–	–	5.83	2.86	11.72	2.60	1.07	7.31
iPerceive DVC [41]$^\dagger$	–	–	$\mathbf{6.13}$	$\mathbf{2.98}$	12.27	2.93	1.29	7.87
Lu et al. [44]$*$	–	–	6.04	2.78	11.79	3.01	1.31	7.34
CM	–	–	4.69	2.19	11.08	$\mathbf{3.98}$	1.84	$\mathbf{8.93}$

The results are reported on the validation subset of ActivityNet Captions in both settings: captioning ground truth (GT) and learned proposals on BLEU@3-4 (B@3-4) and METEOR (M) metrics. “RL” indicates whether the reinforcement learning is used for training, and “FD” indicates whether the model is trained on the complete ActivityNet Captions dataset. “$*$” means that a single video modal data is used, and “$^\dag$” means that a cross-modal data is used. The best results are highlighted

In Table 3, when viewed from “GT Proposals”, although B@3, B@4 and M of CM are in the upper middle level among state-of-the-art models, there is a gap with iPerceiveDVC. When viewed from “Learned Proposals”, except that B@4 is slightly lower than BMT, CM outperforms all of the models. The reason for the different results is that CM was designed to solve the problems of inaccurate localization of events and redundant parameters in the models, not to improve the performance of generating captions. When the model needs to localize proposals, the advantages of CM become apparent. We conducted the following experiments to quantify the advantages of CM in terms of localization events and the number of parameters.

**Fig. 15:**
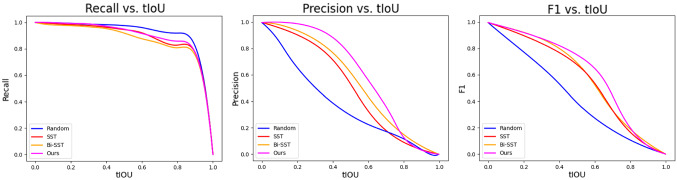
Performance change of proposal generation methods with different *tIOU* thresholds

**Localize events:**

**Table 4:** Comparison of localizing events with state-of-the-art models on dense video captioning task

Method	FD	Prec	Rec	F1
Xiong et al. [41]	$\checkmark$	51.41	24.31	33.01
Mun et al. [27]	$\checkmark$	$\mathbf{57.57}$	55.58	56.56
Zhou et al. [42]	$\checkmark$	38.57	86.33	53.31
Wang et al. [9]	$\checkmark$	44.80	57.60	50.40
Iashin et al. [20]	–	45.90	87.51	60.21
BMT [43]	–	48.23	80.31	60.27
iPerceive DVC [41]	–	44.79	82.30	58.01
Lu et al. [44]	–	54.77	63.59	58.85
CM	–	49.10	$\mathbf{88.32}$	$\mathbf{63.11}$

The best results are shown in bold

Metrics: precision, recall, and f1-score

In the experiments, we set the event proposal generator’s *tIOU* thresholds to 0.3, 0.5, 0.7, and 0.9. Table 4 shows the metrics’ average values under the four *tIOU* thresholds. The precision of the event proposal generator is comparable to that of the Mun et al. and Xiong et al. methods, but our model uses less data. In terms of other metrics, CM outperforms other models in event localization.

In addition, we investigated the performance variation of various proposal generation methods under different *tIOU* thresholds. Since most of the mainstream DVC models use Random, SST [21] and Bi-SST [9] to localize events, we choose them to complete this experiment as well. As shown in Fig. 15, considering the three metrics together, CM has more advantages than other methods in localizing events.

**Parameters:**

**Table 5:** Comparison with other state-of-the-art methods in terms of parameters

Method	Parameters (million)
Iashin et al. [20]	149.70
BMT [43]	54.92
iPerceive DVC [41]	158.37
CM	$\mathbf{53.55}$

The best results are shown in bold

To get a clear idea of the number of parameters in the models, we count the parameters of CM and compare them with other state-of-the-art models. As can be seen from Table 5, CM has the least number of parameters among the mainstream DVC methods based on deep learning. It only has roughly 53.55 million parameters, which is about 1.37 million less than BMT. This result demonstrates that CM is a lightweight model with fewer parameters.

#### Ablation study

**Table 6:** The impact of training procedures and input modal data

Training procedure	Modality	GT Proposals			Learned Proposals		
		B@3	B@4	M	B@3	B@4	M
	Audio	2.09	0.93	7.36	2.27	0.73	6.33
Separately	Visual	3.86	1.60	10.37	2.84	1.24	7.08
	Cross-modal	4.69	2.19	11.08	2.94	0.93	8.82
Proposal	Audio	1.78	0.76	7.22	1.90	0.71	5.69
$\rightarrow$	Visual	3.63	1.60	10.23	2.05	0.74	6.14
Captioning	Cross-modal	4.13	1.75	10.36	2.91	1.44	6.91
Captioning	Audio	2.09	0.93	7.36	1.95	0.96	6.24
$\rightarrow$	Visual	3.86	1.60	10.37	3.01	1.23	7.63
Proposal	Cross-modal	4.69	2.19	11.08	3.98	1.84	8.93

To illustrate the impact of multi-modal data and training procedure on CM, we employ ablation experiments to disassemble the modal data and training procedure. In Table 6, “Separately” means that the event proposal generator and the caption generator are trained independently and do not share the cross-modal encoder. “Proposal$\rightarrow$ Captioning” means that the event proposal generator is trained first, and then the cross-modal encoder trained in the first stage is used to train the caption generator. “Captioning$\rightarrow$ Proposal” is the opposite task.

Analyzed from the perspective of the training procedure. When evaluated through the perspective of “GT Proposals”, “Separately” and “Captioning$\rightarrow$ Proposal” perform similarly. From “Learned Proposals”, “Captioning$\rightarrow$ Proposal” performed better. Therefore, the training procedure of all experiments is carried out with “Captioning$\rightarrow$ Proposal”.

Analyzed from the perspective of different modalities, for single-modality data, the metrics obtained using visual modality are much better than those obtained using audio modality. However, when compared to cross-modal data, the visual modality is at a disadvantage. Overall, regardless of the training procedure, cross-modal data has advantages over single-modality data, indicating that cross-modal data can be helpful for generating captions.

#### Qualitative results

**Fig. 16:**
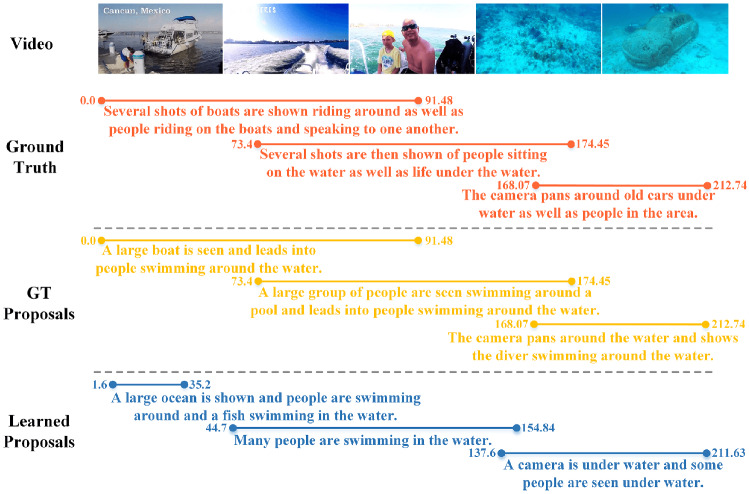
Qualitative samples of CM. “GT proposal” means that we generate a caption on the premise that we know the timestamp of event. “Learned Proposals” means that we don’t know the timestamp

In Fig. 16, based on the qualitative results of the video, we can conclude that our model is reasonable in localizing events and the generated captions are logical.

### Results of CMCR

In this section, we compare CMCR with CM and other state-of-the-art models to analyze the impact of CR on CM in generating captions.

#### Experiment details

In visual prior knowledge extractor, we select the pre-trained ResNet-101 as the feature extractor the pre-trained Mask R-CNN [33] to obtain the bounding box of the object, and the stochastic gradient descent (SGD) [45] as the optimizer. And for training, we set the batch size to 4 and the initial learning rate to 0.0005. The learning rate decays at a rate of 10 $\times$ from the 50th epoch, and 240,000 iterations were completed on the MS-COCO dataset using GeForce RTX 3090, for a total of 70 epochs and a total time of 16.8 h.

When training the language prior knowledge extractor, the learning rate was set to 0.0005, decayed by 20% every 5 epochs, and the batch size was set to 64. It took 7.23 h to train 60 epochs on the MS-COCO dataset.

The objective of training the unbiased visual scene graph generator is to enable the model to correctly predict the entity classes, attribute classes, and relationship classes observed in the images. We set the batch size to 12 and the initial learning rate to 0.01. When the model’s performance on the validation set stabilized, the learning rate decreased by a factor of 10. This module was trained on the VG150 dataset for 60 epochs and took 11.6 h.

#### Comparison to the state-of-the-art

**Fig. 17:**
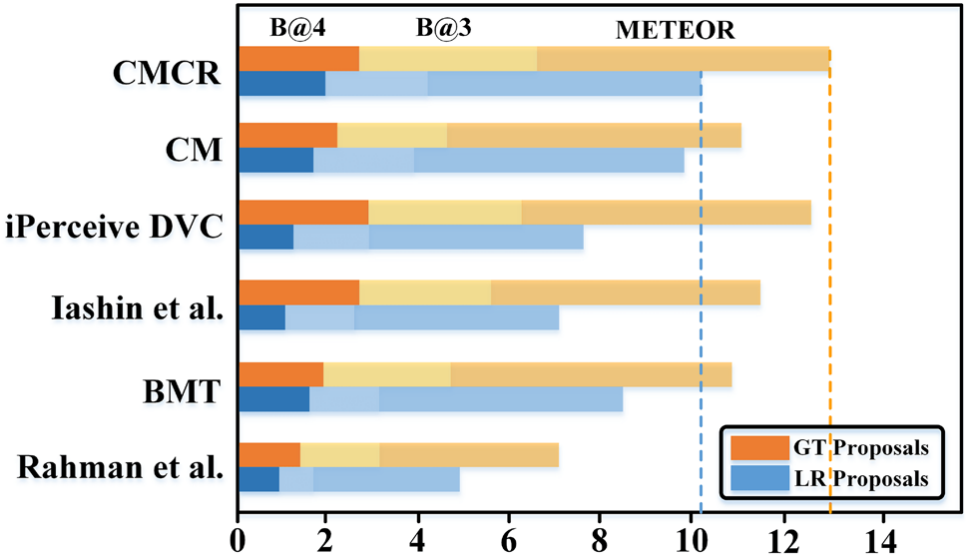
Performance comparison between CMCR and state-of-the-art method

**Table 7:** The performance comparison with the state-of-the-art methods

Method	Parameters (million)	GT Proposals			Learned Proposals		
		B@3	B@4	M	B@3	B@4	M
Rahman et al. [24]	–	3.04	1.46	7.23	1.85	0.90	4.93
Iashin et al. [20]$*$	–	4.12	1.81	10.09	2.31	0.92	6.80
iPerceive DVC [41]$*$	–	5.23	2.34	11.77	2.59	1.07	7.29
BMT [43]	54.92	4.63	1.99	10.90	3.84	1.88	8.44
Iashin et al. [20]$^\dag$	149.7	5.83	2.86	11.72	2.60	1.07	7.31
iPerceive DVC [41]$^\dag$	158.37	6.13	2.98	12.27	2.93	1.29	7.87
Lu et al. [44]$*$	–	6.04	2.78	11.79	3.01	1.31	7.34
CM	$\mathbf{53.55}$	4.69	2.19	11.08	3.98	1.84	8.93
CMCR	67.34	$\mathbf{6.78}$	$\mathbf{3.13}$	$\mathbf{12.98}$	$\mathbf{4.27}$	$\mathbf{2.06}$	$\mathbf{10.09}$

“$*$”Single visual modal data is used

“$^\dag$”Cross-modal data is used. The best results are highlighted

**Table 8:** The effect of different visual knowledge extraction methods on the performance of CMCR

Method	Visual knowledge extraction method	GT proposals			Learned proposals		
		B@3	B@4	M	B@3	B@4	M
CM	–	4.69	2.19	11.08	3.98	1.84	8.93
CMCR	ResNet-101	5.87	2.67	11.55	4.07	1.89	9.23
	ResNeXt-101	6.26	2.98	12.33	4.31	2.11	9.73
	Transformer	6.62	$\mathbf{3.29}$	12.46	$\mathbf{4.36}$	$\mathbf{2.26}$	10.06
	CRvpk	$\mathbf{6.78}$	3.13	$\mathbf{12.98}$	4.27	2.06	$\mathbf{10.09}$

CRvpk extractor is the visual prior knowledge extractor that we propose in the CR module

The best results are shown in bold

**Table 9:** Comparison of using different modal knowledge

Method	Prior knowledge	GT Proposals			Learned Proposals		
		B@3	B@4	M	B@3	B@4	M
CMCR	None	4.69	2.19	11.08	3.98	1.84	8.93
	Visual	6.36	2.70	11.39	4.20	1.99	9.58
	Language	5.27	2.29	11.27	4.03	1.81	9.36
	Cross-modal	$\mathbf{6.78}$	$\mathbf{3.13}$	$\mathbf{12.98}$	$\mathbf{4.27}$	$\mathbf{2.06}$	$\mathbf{10.09}$

The best results are shown in bold

Table 7 shows that in all metrics, CMCR achieves the optimal level in the state-of-the-art models. The number of parameters for CMCR goes up from 53.55 million to 67.34 million, slightly higher than BMT. However, the cost is acceptable compared to the performance improvement. As shown in Fig. 17, we use histograms to visualize the performance of CMCR. All three metrics of CMCR lead in both “GT Proposals” and “Learning Proposals”, indicating that CR can greatly improve the performance of CM in generating captions.

#### Ablation study

**Fig. 18:**
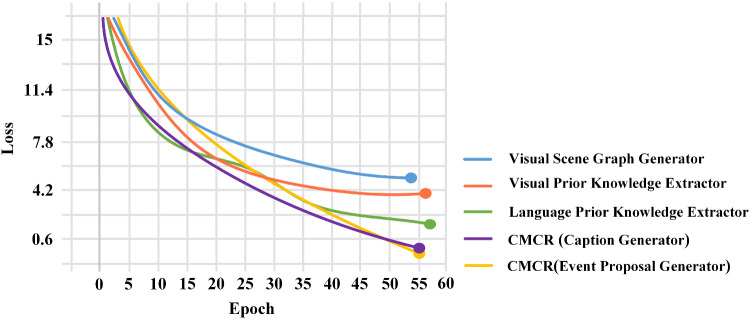
The loss of visual prior knowledge extractor, language prior knowledge extractor, visual scene graph generator, the caption generator of CMCR and event proposal generator of CMCR during the training process

**Fig. 19:**
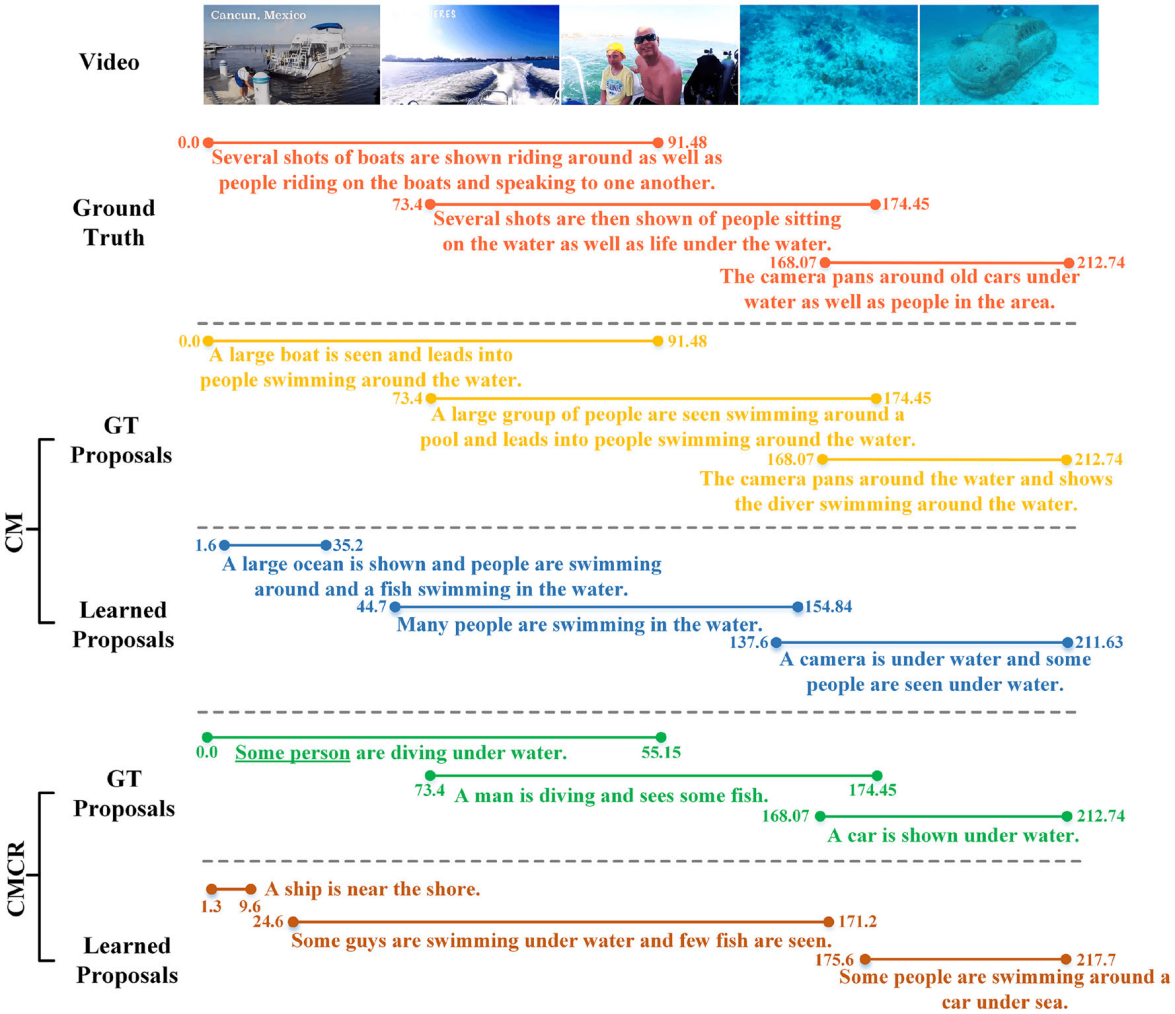
Qualitative samples of CMCR

To explore the superiority of the visual prior knowledge extractor proposed in this paper for association mining, it is compared with other mainstream networks used to extract visual information. In Table 8, CMCR+CRvpk has better performance than CMCR+ResNet-101 and CM+ResNeXt-101, but does not exceed CMCR+Transformer [12]. However, this does not imply that our visual prior knowledge extractor should be replaced by a Transformer.

The specific reasons are as follows, as a generic pre-trained model, Transformer focuses on extracting generic features and is not optimized for mining the causal correlation in the image context. In contrast, CRvpk module uses unbiased scene graph to reason about causal associations between entities, it is superior to Transformer in BLEU@3 and METEOR with much less network parameters. Thus, using our proposed CRvpk to extract visual knowledge is a relatively optimal choice for CR.

To explore the effect of prior knowledge of different modalities on video captions, we report four scenarios in Table 9. When only a single modal prior knowledge is used, the performance of the model is improved, and the improvement brought by visual prior knowledge is more obvious. When using cross-modal prior knowledge, it achieves the best performance, demonstrating that multi-modal prior knowledge benefits in the generation of captions.

In order to intuitively understand the convergence of each module and evaluate their training difficulty, we visualize the loss of modules of CMCR during training, as shown in Fig. 18 (the curve has been smoothed).

On the whole, although multiple modules are involved in CR, the modules can complete convergence within a predictable time. Therefore, it is feasible to train CR and embed it into CM.

#### Qualitative results

Finally, we compare the qualitative results of CMCR and CM, as illustrated in Fig. 19. After embedding CR into CM, the localization of events and generated captions have changed, but it is difficult to distinguish positive changes or negative changes only from the qualitative results. However, by embedding CR, we can be certain that CMCR can reason about the overall behavior, entity, or scene based on the existing entities and relationships. And the captions with grammatical errors are underlined. This is something we should address in our future research.

## Conclusion

In this paper, we proposed a novel DVC model named CMCR, which is mainly composed of CM and CR, to improve event localization and exploit semantic relationships among entities with reduced network parameters. In CM, we use a cross-modal attention fusion layer to fuse multi-modal data. By reusing the cross-modal encoder, the number of parameters in the model is greatly reduced. Additionally, our proposed event refactoring algorithm ensures that the captions have both temporality and comprehensiveness. In CR, we use knowledge-enhanced unbiased scene graphs to reason about causal relationships between entities. We perform our experimentation on the ActivityNet Captions dataset and achieve state-of-the-art results. And the captions generated by CMCR are more reasonable and contain more information. In future research, we will check the grammar of captions and optimize the cross-modal encoder and decoder to reduce the probability of generating grammatical errors.
